# Evaluation of syringin’s neuroprotective effect in a model of neonatal hypoxic-ischemic brain injury

**DOI:** 10.55730/1300-0144.5697

**Published:** 2023-06-21

**Authors:** Ezgi Yangın ERGON, Aslı ÇELİK, Gülden DİNİZ, Rüya ÇOLAK, Senem Alkan ÖZDEMİR, Şebnem ÇALKAVUR, Osman YILMAZ

**Affiliations:** 1 Neonatal Intensive Care Unit, Pediatric Division, Dr Behçet Uz Children’s Education and Research Hospital, İzmir, Turkiye; 2 Department of Laboratory Animal Science, Faculty of Health Sciences, Dokuz Eylül University, İzmir, Turkiye; 3 Department of Pathology, Medical Faculty, İzmir Democracy University, İzmir, Turkiye; 4 Neonatal Intensive Care Unit, Pediatric Division, Medikal Park Florya Hospital, Aydın University Medical Faculty, İstanbul, Turkiye

**Keywords:** Hypoxic-ischemic, brain injury, antiinflammatory, apoptosis, syringin

## Abstract

**Background/aim:**

A significant cause of mortality and morbidity in the neonatal era is hypoxic-ischemic encephalopathy (HIE). This study examined the histopathological analysis and neuroprotective impact of syringin (SYR) in an experimental HIE rat model.

**Material and methods:**

On the 7th postnatal day, 24 Wistar albino rats were evaluated in 3 groups using the HIE model under gas anesthesia. In the experiment, Group A received 10 mg/kg SYR plus dimethyl sulfoxide (DMSO), Group B received DMSO only, and Group C served as a sham group. Immunohistochemical techniques were used to assess apoptotic cell measurement and proinflammatory cytokines (TNF-α and IL-1β primary antibodies).

**Results:**

Rats suffering from hypoxic-ischemic brain damage had their apoptosis assessed. The SYR and sham groups had statistically fewer cells undergoing apoptosis (p < 0.001). There was no difference between the groups in terms of IL-1β and TNF-α during immunohistochemical staining. Neuronal degeneration was significantly lower in the histological evaluation of the hippocampus in the SYR group (p = 0.01). A statistically significant difference (p = 0.01) was observed between the SYR and the control groups regarding pericellular and perivascular edema.

**Conclusion:**

SYR reduced apoptosis, perivascular and pericellular edema, and neuronal degeneration in rat cerebral tissue. These results raise the possibility that SYR may have a neuroprotective effect on the harm brought on by HIE. This is the first investigation of SYR’s function within the HIE paradigm.

## 1. Introduction

The mortality and morbidity rates of neonatal encephalopathy (NE) are significant. Hypoxic-ischemic encephalopathy (HIE), prenatal infections, aberrant placentas, metabolic problems, coagulopathies, and newborn vascular stroke are several causes of NE. Neonatal HIE is the term used to describe brain damage brought on by low blood flow (ischemia) and insufficient oxygen supply (hypoxia) during the perinatal period, which may result in early demise or a number of morbidities later in life, such as cerebral palsy, epilepsy, cognitive impairment, and developmental delays in the motor and nervous systems [1,2]. Approximately 1 million infants die each year from HIE [3]. According to a country’s development level, the newborn HIE incidence rate varies; in industrialized countries, the HIE rate is 1–2 per 1000 live births, but in developing countries, the rate is 10–20 per 1000 live births [4,5].

Apoptotic cell death in neurons occurs within hours or days of the initial insult in neonatal HIE, which has three distinct phases. Therapeutic hypothermia (cooling the entire body to 33.5 °C for 72 h, beginning within the first 6 h after delivery) is the only neuroprotective treatment with scientifically demonstrated efficacy in newborn HIE to date, and it works in phases I and II [6,7]. At 18 months old, this treatment increased survival with better neurological results [7].

The occurrence of adverse effects following hypoxia-ischemia has been investigated in relation to a number of neuroprotective therapies, including erythropoietin, melatonin, cyclooxygenase inhibitors, and glutamate receptor antagonists [8,9]. There is still no consensus about medical treatment options.

Eleutheroside B [10], also known as Ciwujia or Siberian ginseng, is a lignan phenolic molecule found in Acantopanax plants called syringin (SYR), used as an adaptogen to control adrenal gland function and help the body cope with stress [11]. SYR produces a neuroprotective effect by lowering inflammatory markers (TNF-a, IL-6, and IL-1β expression), infarct size, and cerebral edema [12]. In experimental rat models, this substance has been shown to produce a wide range of pharmacological activities, such as antiinflammatory, antioxidant, hepatoprotective, and renoprotective effects [13]. Additionally, it is efficient in lowering inflammation in brain ischemia/reperfusion injuries in rats in recent studies [14,15].

In the present study, we aim to evaluate the neuroprotective effect of SYR in the early period of perinatal asphyxia by reducing inflammatory factors, cytokines, and apoptosis in an HIE rat model.

## 2. Materials and methods

### 2.1. Animals

The study involved 24 Wistar albino baby rats, which ranged in weight from 9 to 13 g. The Multidisciplinary Laboratory Experimental Animals Unit of Dokuz Eylül University (DEU) provided the rats. The rats were kept in well-ventilated spaces with 12-h light/dark cycles, relative humidity levels between 40%–60%, and a room temperature between 20 °C–24 °C. The loudest it could get in their living spaces was 85 dB. The rodents were given ad libitum access to drinking water, conventional rat pellets, and litter in their natural surroundings. Cannibalism was avoided by manipulating the baby rats as much as possible with cotton by lab monitors wearing gloves. The Animal Experiments Local Ethics Committee approved the study (approval No. 63/2021). The animals were treated in accordance with the guidelines of the ethics committee.

### 2.2. Establishment of hypoxic-ischemic brain injury

#### 2.2.1. Anesthesia administration and surgical process

A modified Levine–Rice process was modeled for the study’s design [16]. For surgical procedures, sevoflurane was used as anesthesia. Neonatal rats were supine under general anesthesia, and a midline neck incision was made. The trachea was found during the dissection under the microscope, the left common carotid communis was fixed and permanently ligated with 5/0-silk sutures, and the subcutaneous tissue and skin were cleansed and closed with sutures. It took no more than 7 min to complete the surgical procedure. The study did not include rats that developed respiratory arrest due to anesthesia or bled heavily due to ligation.

Each experimental animal was housed in a glass jar with a volume of 450 mL and a gas inlet and output system. The glass jars were filled with humidified 92% pure nitrogen and 8% oxygen. A Draeger anesthetic gas monitor was utilized to track the hypoxic combination. The newborn rats were placed in a 37 °C water bath and allowed to relax for 1–2 h before being exposed to the hypoxic mixture for 2.5 h. The treatment, dimethyl sulfoxide (DMSO) solution, was administered to the groups. The rats were sacrificed under high-dose ether anesthesia 24 h after treatment. The complete extracted brain tissues were taken for pathological analysis after the rats had been sacrificed. The removed tissues were fixed in 10% neutral buffered formaldehyde. After fixation, all brains were examined in parallel sections in the coronal plane. During histopathological examination, the right sides were stained with blue surgical margin dye to distinguish the right and left sides of the brains.

#### 2.2.2. Reagents

SYR was supplied by ChemFaces (98% purity; ChemFaces, Wuhan, China, 118-34-3). SYR has a solid structure and needs a solvent to activate. According to the manufacturer’s instructions, DMSO was used as a solvent for SYR. There is no known precise dose of SYR for neonatal rats. However, SYR’s neuroprotective effect in cerebral ischemia in adult rat models has previously been studied, and 3 different doses have been used: 5, 10, or 20 mg/kg/doses. Among these doses, SYR was ineffective on infarct volume when administered at 5 mg/kg/dose, while 10 and 20 mg/kg/doses were effective. For this reason, the minimum drug dose in which SYR is effective was taken as a sample for our study, and 10 mg/kg of this solution was then injected into the intraperitoneal cavity of the rats [14].

#### 2.2.3. Grouping

Twenty-four participants were chosen as the study’s sample, with a power of 90% and a two-sided error of 5% [17]. Three groups of 8 rats each were created for the experiment.

### 2.3. Pathological examination

#### 2.3.1. Tissues sampling

Hematoxylin and eosin (H and E) staining was applied to tissues removed from the formalin solutions after paraffin was initially applied to the tissues. An expert pathologist analyzed the histological alterations using an Olympus BX51 light microscope, which may be captured with an Olympus DP72 camera. Necrosis, lymphocyte infiltration into the interstitium, and vascular, neuronal, and glial cell alterations were considered while scoring. A visible change received a score of (0), little or slight alteration (1), moderate change (2), and severe change (3). While creating the study model, the left carotid artery was blocked, and hypoxia was created. In this model, the left cortex was mainly affected. Therefore, during statistical analyses, we evaluated only the left lobes of the brain.[Table t2-turkjmedsci-53-5-1312]

**Table t2-turkjmedsci-53-5-1312:** 

Groups	Group name	Number of rats	Anestheia	Hypoxic ischemic brain injury model	Substance usage (single dose: 0.2 mL)
Group A	experimental group	8	+	+	DMSO + SYR*
Group B	positive control group	8	+	+	DMSO
Group C	sham	8	+	−	−

* 10 mg/kg per pup (0.1 mg for a 10-g rat) (14)

#### 2.3.2. Immunohistochemical evaluation

The apoptosis assay kit was used to investigate the terminal deoxynucleotide transferase dUTP labeling (TUNEL) approach, which finds DNA fragmentation in the cell nucleus during apoptosis (TUNEL Chromogenic Apoptosis Detection Kit Catalog No. A049). After deparaffinization in xylene, tissue sections were hydrated using a series of ethanol solutions. After 30 min at room temperature in the presence of 20 g/mL of proteinase K, the sections were washed in dH2O. Incubation with 3% hydrogen peroxide in phosphate-buffered saline (PBS) for 15 min at room temperature reduced endogenous peroxidase activity. The sections were exposed to a TdT-enzyme for 120 min at 37 °C in a humid environment after being treated with equilibration buffer for 3–5 min; they were then incubated for 60 min with an antidigoxigenin compound after being heated vigorously for 10 min in a stop/wash buffer at room temperature. The sections were manually boiled in a pH 6.0 citrate solution at 65 °C for 20 min after being cut into slides with lysine for immunohistochemical staining and deparaffinized at 60 °C for 3 h. Using the biotin-avidin technique, the slides were incubated with primary antibodies for an hour at room temperature after cooling in a buffer solution. The steps were divided using PBS washes. After counterstaining with hematoxylin and labeling with a peroxidase substrate, the slices were dried, cleaned, and mounted. The TUNEL-positive cells in high-power fields (HPF) were counted, and the percentage according to cell count was determined. To evaluate the percentage of apoptotic cells, at least 10 microscopic HPFs in each slide were examined, the percentages of stained cells were accounted for, and the mean value was established.

#### 2.3.3. IL-1β and TNF-α measurement in tissue

Cerebral tissues were manually immunohistochemically stained with primary antibodies against interleukin-1β and TNF-α. Kits were used to test interleukin-1β (polyclonal; Wuhan Fine Biotech Co., China; 1/100 dilution; catalog number: FNab10305) and TNF-α (polyclonal; Wuhan Fine Biotech Co., China; 1/50 dilution; catalog number: FNab08821). For immunohistochemistry staining, the best paraffin block for evaluation was selected. After deparaffinization, the slices were immersed in EDTA solution (pH: 8.0), heated for 20 min, and allowed to cool in buffer solution for 5 min to prepare them for immunohistochemistry staining. Slides were then incubated at room temperature with primary antibodies using avidin-biotin techniques. Intense membranous or cytoplasmic staining for these two antibodies was graded as +3 and light staining as +1 in all neuronal cells [18].

### 2.4. Statistical analysis

SPSS 25.0 (IBM Corp., Armonk, New York, USA) was used to analyze the variables. The Shapiro–Wilk test determined whether all data were normally distributed. The analysis of parametric data utilized the analysis of variance (ANOVA) test because the distribution of the groups was homogeneous. In the one-way ANOVA test, the groups were compared using Levene’s homogeneity test. When there was a discrepancy in the post hoc analysis in the ANOVA for homogeneous variables, Bonferroni post hoc analysis was used. The mean and standard deviation of the data were displayed. The Kruskal–Wallis test was employed in the triple data analysis of the groups when parametric analysis could not be completed. A statistically significant difference between the categories was defined as p < 0.05.

## 3. Results

In the hypoxic environment, 3 rats from the treatment group and 1 rat from the DMSO group perished, while 2 rats from the sham group died due to cannibalism. As a result, the study included 5 rats in the treatment group, 7 in the DMSO group, and 6 in the sham group. There was no difference between the groups in terms of weight or gender. The mean body weight of groups was 12.0 ± 1.0 g in Group A, 12.4 ± 1.3 g in Group B, and 11.3 ± 3.2 g in Group C (p = 0.65).

With the help of the immunohistochemical TUNEL method, the apoptosis of rats with hypoxic-ischemic brain injury was evaluated. The number of cells undergoing apoptosis in the SYR and sham groups was found to be statistically significantly lower (p < 0.001) (Table). Fifty-one apoptotic cells were detected in the SYR group, 47.3 in the sham group, and 91.8 in the control group. As shown in Figure 2, the right and left brains were histopathologically compared in both cerebral cortexes.

**Figure 1:**
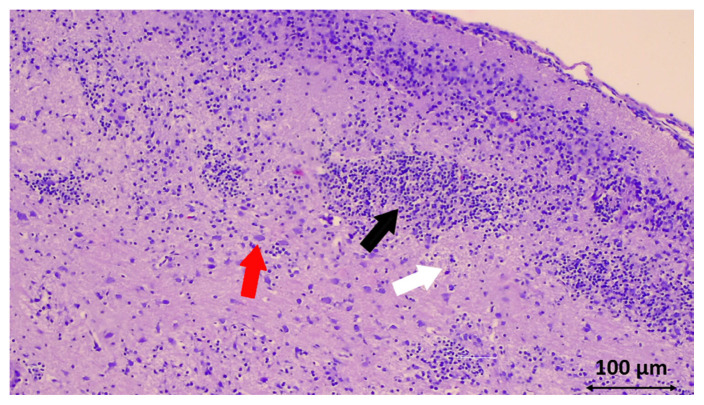
Cortical region of the cerebral occipital lob. Note the damaged reddish fuzzy-shaped neurons (red arrow), pericellular and perivascular edema (white arrow), increasing glial cells, and microglial nodules (black arrow) in a rat inof the control group

**Figure 2:**
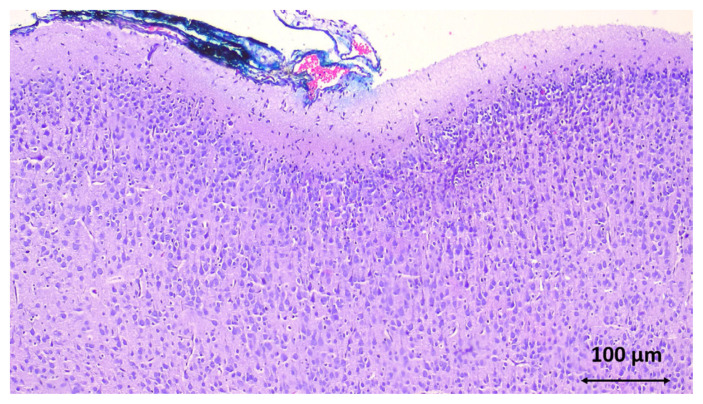
Cerebral cortex of the rats. The right cortex was stained blue (HE × 40).

**Figure 3:**
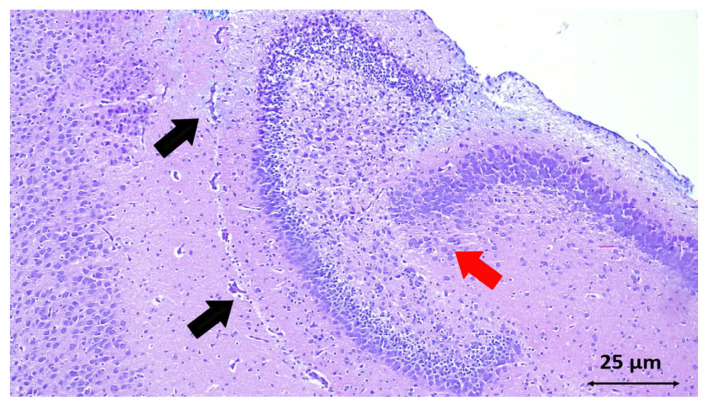
Haematoxylin Eosin staining of the Hippocampal Cornu Ammonis region in the control group., Note the damaged reddish neurons (red arrow), congestion, and perivascular edema (black arrows) (HE × 40).

**Figure 4:**
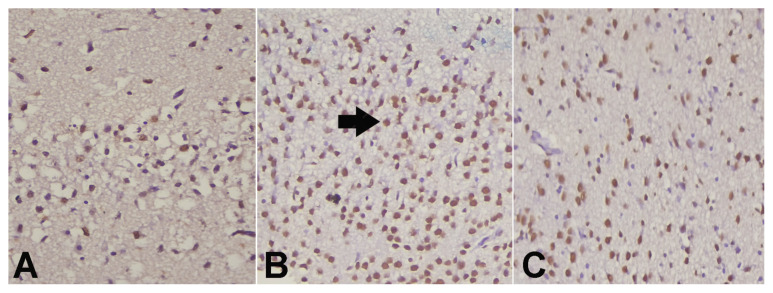
Apoptotic cells exhibiting DNA fragmentation in the nuclei in the cerebral cortex of the rats: A) Syringin group;, B) Control group;, and C) Sham group (TUNEL staining × 200).

Histological analysis of the hippocampus revealed a substantial reduction in neuronal degeneration in the SYR group (Table and Figure 3) (p = 0.01). Although there was less neuronal degeneration in the cortex in the SYR group, there was no statistically significant difference between the groups **(**Figure 1) (p = 0.07). The apoptosis rate is compared in Figure 4 for each of the 3 experimental groups.

**Figure 5:**
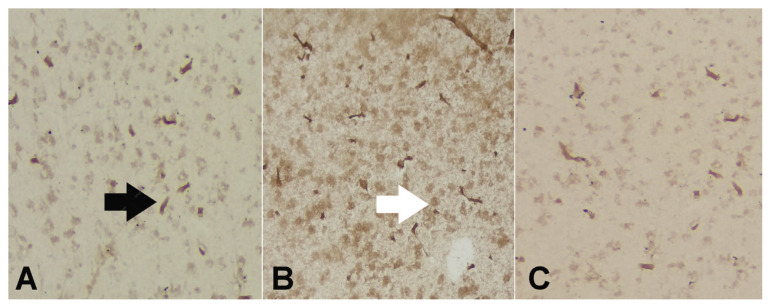
IL-1β expressions of mainly endothelial cells (black arrow) and some neurons and glial cells (white arrow): A) SYR group with low IL-1β expressions;, B) Control group with high IL-1β expressions; and C) Sham group with low IL-1β expressions (DAB × 200).

**Figure 6:**
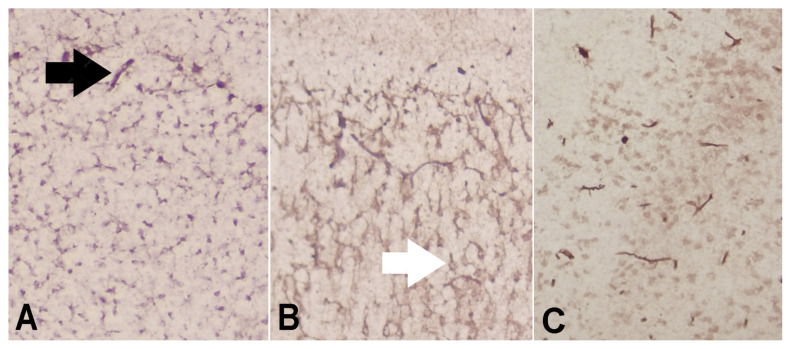
TNF-α expressions of mainly endothelial cells (black arrow) and some neurons and glial cells (white arrow): A) SYR group;, B) Control group,; and C) Sham group (DAB × 200).

IL-1 β and TNF-α are two common proinflammatory cytokines associated with neuroinflammation. Immunohistochemical staining revealed no differences in the IL-1β and TNF-α levels between groups, as shown in Table and Figures 5 and 6 (p = 0.18, p = 0.39).

Glial cells and microglial nodules were similar between the groups (p = 0.27). Pericellular and perivascular edema were detected less in the SYR group, constituting a statistically significant difference (Table) (p = 0.01).

## 4. Discussion

A neonatal HIE rat model was used for the current investigation to examine the neuroprotective function of SYR based on its previously identified pharmacological effects. We showed that SYR reduces the number of apoptotic cells, cerebral edema, and neuron damage in the cerebral tissue of rats.

Untreated neonatal HIE creates a heavy burden that may negatively affect families financially and emotionally from their child’s infancy to maturity. It has also emerged as a leading medical problem worldwide. Many scientists have conducted numerous animal tests and clinical studies because of the medical importance of neonatal HIE. The only proven neuroprotective therapy used in neonatal HIE clinics is therapeutic hypothermia. Many preclinical studies on neuroprotective therapy are ongoing, and newly discovered agents are being evaluated as candidates and supportive treatments for neuroprotective therapy in the clinical setting.

Animal studies demonstrate that phases I, II, and III can be used to categorize the pathophysiological alterations in neonatal HIE. Disruption of cerebrovascular autoregulation and the resulting primary and secondary energy deficiency are responsible for the pathogenesis of cerebral injury. Furthermore, pathophysiological changes may persist for days or even weeks. The reperfusion period following HIE is the most critical stage of the disease when the most severe injury can occur. Before this reperfusion period, treatment may stop or reduce apoptosis. After 6–72 h of hypoxia-ischemia, the body enters phase II (secondary energy failure phase), during which excitatory neurotransmitters and free radicals are still generated, mitochondrial dysfunction worsens, and P stores are exhausted [9]. In addition, inflammatory factors come into play, and brain damage becomes more pronounced [19]. Available neuroprotective medicines for the early treatment of neonatal hypoxic-ischemic brain injury are now divided into 4 categories based on known processes, although they are not yet proven as therapy options: antiapoptosis, antioxidation, antiinflammatory, and antiexcitotoxicity [8,20]. SYR, which we believe to be a new neuroprotective agent in the HIE model, targets phases I and II of the process and uses its antiapoptotic impact to attempt to stop it with the minimum amount of harm possible.

SYR (eleutheroside B) is a natural chemical compound first discovered by Meillet in 1841 and isolated from the bark of the lilac (*Syringa vulgaris*). It has been detected in many plant species until today. Chemically, sinapyl is a glycoside of the alcohol-phenylpropanoid glycoside compound [21,22]. To date, its many pharmacological effects have been assessed and described. SYR is an antioxidant; Strawa et al. and Kim et al. found that it has a radical scavenging effect on 1,1-diphenyl-2-picrylhydrazil (DPPH) [23] and an inhibitory effect on nitric oxide synthesis [24]. SYR is protective against neuronal cell damage, and in Liu’s study, it reduced inflammation and cerebral damage in rats with cerebral ischemia/reperfusion injury [15]. SYR has antiapoptotic effects due to decreased caspase-3 activation [25]. Moreover, SYR’s antidiabetic, antiulcer, antiinflammatory, and immunomodulatory effects are well documented [13,26,27]. Many studies exist about the additional effects of SYR, but no information on its impact on HIE was found.

Anaerobic fermentation of glucose occurs in the early stages of hypoxic-ischemia injury, lowering ATP levels and causing a number of cascades, primarily pericellular and perivascular edema, in neuronal cells. Pericellular and perivascular edema are two of the most significant predictors of intense inflammation in that area. Whether many antiinflammatory molecules have a reducing effect on cerebral edema has been previously evaluated in the literature. In an adult-based cerebral ischemia model, SYR significantly decreased infarct size and cerebral edema and enhanced rat neurological functions [14]. Similarly, in our study, SYR decreased pericellular and perivascular edema in the neonatal HIE model.

Cellular necrosis and apoptosis caused by ischemia and reperfusion injury begin in phase II of neonatal HIE and persist for long periods. To date, evaluations have been conducted on whether many neuroprotective molecules, such as humic acid and pycnogenol, have apoptotic effects in a hypoxic-ischemic brain injury model [18,28]. Similarly, administration of SYR in the therapeutic dose range may reduce apoptosis in hypoxic-ischemic brain injury. Indeed, our study found significantly less apoptosis in the SYR group than in the control group. In another study (Zhang’s cell culture research on Alzheimer’s disease) on SYR and its effect on apoptosis, the authors demonstrated that Aβ_25–35_ decreased cell viability and caused cell death and established that SYR therapy increased cell viability and inhibited cell death in response to Aβ_25–35_ -induced damage in SK-N-SH and SK-N-BE cells [29].

A key activator of inflammatory genes is nuclear factor kappa B (NFκ-B) [30]. Increased NF-κB upregulation results from cerebral tissue suffering from ischemia-reperfusion damage [31]. NF-κB activation induces the expression of several proinflammatory proteins, including IL-1β, IL-6, and TNF-α, which can lead to neuronal death [32]. Previous research using HIE rat models has shown that several compounds exhibit antiinflammatory properties by lowering TNF-α and IL-1β levels via the NF-κB pathway [28]. In the cerebral ischemia-reperfusion model used to study it, SYR, like other compounds, reduced the expression of NF-κB, IL-1β, IL-6, and TNF-α. It also blocked the nuclear translocation of NF-κB [14]. However, in our study, SYR did not affect the immunohistochemical measurements of IL-1β and TNF-α in cerebral tissue. We only used manual immunohistochemical staining to assess IL-1β and TNF-α levels and did not use ELISA. In addition, we did not observe the NF-B pathway for these proinflammatory cytokines. Although SYR’s ability to reduce cerebral edema suggests that it is antiinflammatory, the fact that we only measured IL-1β and TNF-α levels immunohistochemically is a limitation of our study. In future studies on the SYR and HIE models, SYR’s antiinflammatory effect can be evaluated using additional methods.

## 5. Conclusion

This study evaluated how SYR affected the HIE model, and the effects of SYR on an experimental rat model of HI brain injury were examined for the first time. We showed that the SYR group had much less apoptosis, perivascular and pericellular edema, and neuronal degeneration. Given the results, SYR may be a new, inexpensive, and accessible neuroprotective agent in treating HIE.

## Figures and Tables

**Table:** Comparison of groups in terms of neuron damage.

	SYR Group n: 5	DMSO Group n: 7	SHAM Group n: 6	p-values		

	Group A	Group B	Group C	A^®^B	B^®^C	A^®^C

**Tunnel positive apoptotic cell counts** mean ± SD	51 ± 6.5	91.8 ± 10.9	47.3 ± 7.3	<0.000^a^	<0.000^a^	1

**IL-1 β, n**						
Few	-	-	-			
Medium	4	2	4			
Severe	1	5	2	0.185^b^		

**TNF-α, n**						
Few	-	-	-			
Medium	4	5	6			
Severe	1	2	-	0.397^b^		

**ND, hippocampus, n**						
Few	1	-	4			
Medium	3	3	2			
Severe	1	4	-	0.015^b^		

**ND, cortex**, n						
Few	1	-	5			
Medium	2	3	1	0.071^b^		
Severe	2	4	-			

**GMN**, n						
Few	-	3	4			
Medium	1	1	1			
Severe	3	3	1	0.278^b^		

**PS and PV edema**, n						
Few	1	-	4			
Medium	4	4	2			
Severe	-	3	-	0.013^b^		

**Abbreviations:** GMN: gliosis microglial nodule; IL: interleukin; ND: neuron degeneration; PS: pericellular; PV: perivascular; SD: standard deviation; TNF: tumor necrosis factor.

a Analysis of variance test (Bonferroni).

b Kruskal–Wallis test.

## Abbreviations

NE: Neonatal encephalopathy
HIE: Hypoxic-ischemic encephalopathy
SYR: Syringin
DEU: Dokuz Eylül University
DMSO: Dimethyl sulfoxide
H and E: Hematoxylin and eosin
TUNEL: Terminal deoxynucleotide transferase dUTP labeling
PBS: Phosphate-buffered saline
ACOG: American College of Obstetricians and Gynecologists
ATP: Adenosine triphosphate
NF-κB: Nuclear factor kappa B
